# The caecal microbiota promotes the acute inflammatory response and the loss of the intestinal barrier integrity during severe *Eimeria tenella* infection

**DOI:** 10.3389/fcimb.2023.1250080

**Published:** 2023-08-23

**Authors:** Florian Tomal, Guillaume Sadrin, Pauline Gaboriaud, Edouard Guitton, Laura Sedano, Nathalie Lallier, Christelle Rossignol, Thibaut Larcher, Elodie Rouille, Mireille Ledevin, Rodrigo Guabiraba, Anne Silvestre, Sonia Lacroix-Lamandé, Catherine Schouler, Fabrice Laurent, Françoise I. Bussière

**Affiliations:** ^1^ INRAE, Université de Tours, UMR ISP, Nouzilly, France; ^2^ INRAE, UE PFIE, Nouzilly, France; ^3^ INRAE, Oniris, PAnTher, APEX, Nantes, France; ^4^ Laboratoire IHP VETO, Nantes, France

**Keywords:** *Eimeria tenella*, microbiota, germ-free, chicken, inflammation, bacterial translocation

## Abstract

**Introduction:**

Coccidiosis, a disease caused by intestinal apicomplexan parasites *Eimeria*, is a threat to poultry production. *Eimeria tenella* is one of the most pathogenic species, frequently causing a high prevalence of opportunistic infections.

**Objective:**

The objective of this study is to investigate the role of the microbiota in the pathogenesis of severe *Eimeria tenella* infection.

**Methods:**

We have previously shown that microbiota can promote parasite development. To study the effect of the microbiota on the pathogenesis of this infection, we used an experimental condition (inoculum of 10 000 oocysts *E. tenella INRAE*) in which the parasite load is similar between germ-free and conventional broilers at 7 days post-infection (pi). Thirteen conventional and 24 germ-free chickens were infected. Among this latter group, 12 remained germ-free and 12 received a microbiota from conventional healthy chickens at 4 days pi. Caeca and spleens were collected at 7 days pi.

**Results:**

Our results demonstrated caecal lesions and epithelium damage in conventional chickens at 7 days pi but not in germ-free infected chickens. Administration of conventional microbiota to germ-free chickens partially restored these deleterious effects. At day 7 pi, both infected conventional and germ-free chickens exhibited increased gene expression of inflammatory mediators, including *IL15, IFNγ, TNFα* and the anti-inflammatory mediator *SOCS1*, whereas the inflammatory mediators *CXCLi2, CCL20, IL18, CSF1, NOS2, PTGS2, IL1β, IL6*, the receptor *CCR2*, and the anti-inflammatory mediators *TGFβ1* and *IL10* were upregulated only in infected conventional chickens. Notably, the *IL18, PTGS2* gene expression was significantly higher in the infected conventional group. Overall, the inflammatory response enhanced by the microbiota might be in part responsible for higher lesion scores. Epithelial tight junction protein gene expression analysis revealed a significant upregulation of *CLDN1* with the infection and microbiota, indicating a potential loss of the intestinal barrier integrity.

**Conclusion:**

These observations imply that, during *E. tenella* infection, the caecal microbiota could trigger an acute inflammatory response, resulting in a loss of intestinal integrity. Increase in bacterial translocation can then lead to the likelihood of opportunistic infections. Hence, modulating the microbiota may offer a promising strategy for improving poultry gut health and limiting caecal coccidiosis.

## Introduction

Coccidiosis is a disease caused by *Eimeria*, a type of apicomplexan parasite that colonizes the intestine of various animals. In poultry, *Eimeria tenella* (*E. tenella*) is considered one of the most pathogenic species, with high doses of infection leading to the loss of performances, including body weight, feed intake, feed conversion ratio, hemorrhagic diarrhea, and mortality in severe cases. Prophylaxis of *Eimeria* infection is costly and results in significant economic losses of approximately $13 billion per year worldwide in the poultry industry (Blake et al., 2020).

*E. tenella* proliferates in caecal epithelial cells and causes acute inflammation characterized by a local increase in several immune cells, such as T lymphocytes, natural killer cells, and macrophages (Lillehoj and Trout, 1996). Studies have found that various cytokines and chemokines, including *interleukin (IL)1β*, *IL2*, *IL4*, *IL6*, *IL8*, *IL10*, *IL12*, *IL15, IL17*, and *IL18*, *interferon (IFN)γ*, *transforming growth factor beta* (*TGFβ*)1–4, *tumor necrosis factor alpha* (*TNFα*), and *TNF superfamily 15* (*TNFSF15*) and *lipopolysaccharide-induced TNFα factor* (*LITAF*) are increased in caecal tissues during coccidiosis infection (Lillehoj and Trout, 1996; Laurent et al., 2001; Hong et al., 2006; Kim et al., 2019).

The intestinal epithelium acts as a natural barrier to prevent pathogens from entering and spreading throughout the organism. However, *Eimeria* infection damages the epithelium (Witlock et al., 1975), potentially predisposing the animal to opportunistic entry of pathogens. Coccidiosis has been shown to be a factor for necrotic enteritis, which is associated with the presence of the pathogenic bacterium *Clostridium perfringens* (Helmboldt and Bryant, 1971; Van Immerseel et al., 2004). Studies focusing on intestinal integrity in coccidiosis have found an increase in intestinal permeability and an upregulation of the tight junction proteins, claudin-1 (*CLDN1*), and junctional adhesion molecule-2 (*JAM2*) in a mixed-species *Eimeria* infection (Teng et al., 2020). In *E. tenella* infection, an increase in intestinal permeability at day 5 post-infection (pi) associated with an increase in *CLDN1* and claudin-2 (*CLDN2*) and a decrease in occludin (*OCLN*), E-cadherin and tight junction protein-1 (Pham et al., 2021), reflects an alteration in the integrity of the intestinal barrier, potentially favoring secondary infections by opportunistic pathogens.

*E. tenella* develops in the caeca which is part of the intestine the richest in bacterial abundance and diversity. As a result of *E. tenella* infection, alterations in the microbiota diversity and composition are observed (Wei et al., 2013; Oakley et al., 2014; Qi et al., 2019), with an increase in *Enterobacteriaceae* (Proteobacteria), while non-pathogenic bacteria *Lactobacillus* and *Faecalibacterium* (Firmicutes) are decreased (Macdonald et al., 2017; Huang et al., 2018a). Moreover, during *E. tenella* infection, *Clostridium* is found to be increased (Macdonald et al., 2017; Huang et al., 2018a; Huang et al., 2018b). These changes in the microbiota composition and damage to the epithelium may potentially contribute to the development of opportunistic infections.

In a previous study using a recently developed model of germ-free fast-growing chickens (Guitton et al., 2020), we showed that the microbiota plays a role in the development of the parasite *E. tenella* (Gaboriaud et al., 2020). In the present study, we aimed to study the influence of the microbiota on the physiopathology of *E. tenella* infection by increasing the doses of inoculum to the level where the parasite load was similar in both germ-free and conventional chickens. The results suggest a critical role of the microbiota in promoting the acute inflammatory response and loss of intestinal barrier integrity in *E. tenella* infection, predisposing the animal to opportunistic infections through potential bacterial translocation. Future strategies involving the modulation of the composition of the microbiota may improve coccidiosis prophylaxis.

## Results

### Microbiota plays a major role in the genesis of caecal lesions associated with severe *E. tenella* infection

**Figure 1:**
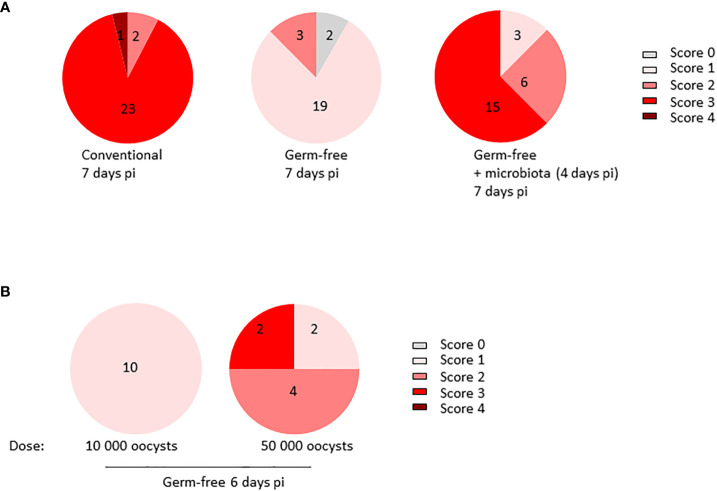
Microbiota plays a major role in the generation of caecal lesions generated by *E. tenella* infection. **(A)** Conventional and germ-free chickens (n ≥ 12/group) were orally infected with *E. tenella* with a fixed dose of inoculum (10 000 oocysts/animal) leading to similar parasite load in germ-free and conventional chickens. At day 4 pi, a part (12 chickens) of infected germ-free chickens received a microbiota from conventional and healthy three-week-old chickens. Lesions were assessed at day 7 pi. Statistical analysis was performed on lesion scores using a Kruskall-Wallis test with a Dunn’s multiple comparisons post-test. Conventional *versus* germ-free: ****P < 0.0001; germ-free *versus* germ-free + microbiota: ****P < 0.0001; conventional *versus* germ-free + microbiota: ns: non significant **(B)** Germ-free chickens were infected for 6 days with a high dose of inoculum (10 000 or 50 000 oocysts/animal; n ≥ 4). Lesion scores were evaluated by the method described by (Johnson and Reid, 1970) and assessed on each caecum (two per animal). Statistical analysis was performed on lesion scores using Mann and Whitney test. **P < 0.01.

To study the intricate interplay between the microbiota and *E. tenella* pathophysiology, we infected ROSS PM3 fast-growing chickens, both germ-free and conventional, with *E. tenella*. Our previous findings revealed that the microbiota significantly influences parasite development and is instrumental in generating caecal lesions. However, to evaluate the contribution of the microbiota on the formation of lesions, we needed to insure similar parasite development in both conventional and germ-free chickens. Following our previous studies (Gaboriaud et al., 2020), we opted for a high inoculum dose of 10 000 oocysts to achieve a similar parasite load at 7 days pi, as evidenced by oocyst counts in the caecal contents of conventional, germ-free, and germ-free chickens supplemented by conventional microbiota (**Supplementary Figure 1**). At day 7 pi, we observed lesions, as described by (Johnson and Reid, 1970). Notably, under these experimental conditions, germ-free chickens had significantly fewer macroscopic lesions than their conventional counterparts. Furthermore, the addition of microbiota from healthy conventional chickens to germ-free chickens at day 4 pi induced the appearance of macroscopic lesions by day 7 pi. These observations suggest that the microbiota contributes to lesion development (**Figure 1A**) regardless of parasite development. Additionally, increasing the inoculum dose to a very high 50 000 oocysts in germ-free chickens resulted in some lesions reaching score 3 in 25% of the animals. Still, the score levels did not match those of conventional chickens receiving 10 000 oocysts (**Figure 1B**). Our results conclusively indicate that microbiota play a critical role in the generation of lesions during *E. tenella* infection.

### Presence of microbiota leads to caecal lesions during *E. tenella* infection and favours mucosal damage

**Figure 2:**
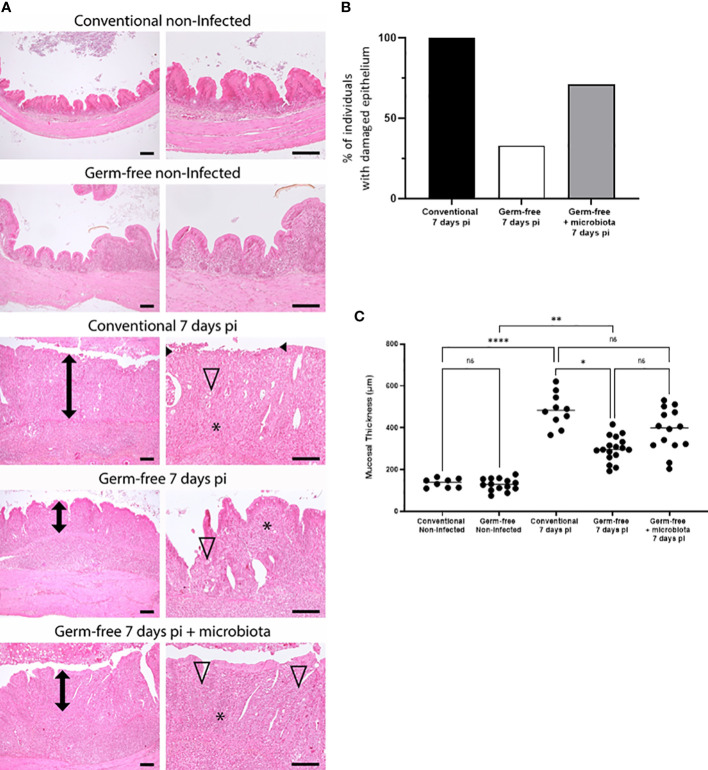
Microbiota favours mucosal damages and increased thickness associated with *E. tenella* infection. **(A)** Mucosal damage was less frequently observed in germ-free *E. tenella* infected chickens compared to conventional infected chickens. Representative histopathological pictures of caeca. Compared to control non-infected animals, infected conventional, germ-free, and germ-free receiving intestinal microbiota animals displayed respectively a severe (ulcerated epithelium indicated into black arrowhead), mild, and marked mucosa thickening (double black arrow). Thickening of the mucosa and submucosa was mainly composed of severe mixed inflammatory cell infiltration (*). *E. tenella* (open arrowhead) were mainly observed within epithelial cells of the mucosa. Hemalun-Eosin-Saffran. Bar=100µm. **(B)** Evaluation of the percentage of animals with epithelium damage (black arrow head) for each infected group. **(C)** Measure of the mucosal thickness (double black arrow) was performed for each animal and are expressed as the median (n ≥ 6/group). Statistical analysis was performed using a Kruskall-Wallis test with a Dunn’s multiple comparisons post-test. *P < 0.05; **P < 0.01; ****P < 0.0001; ns: non significant.

**Figure 3:**
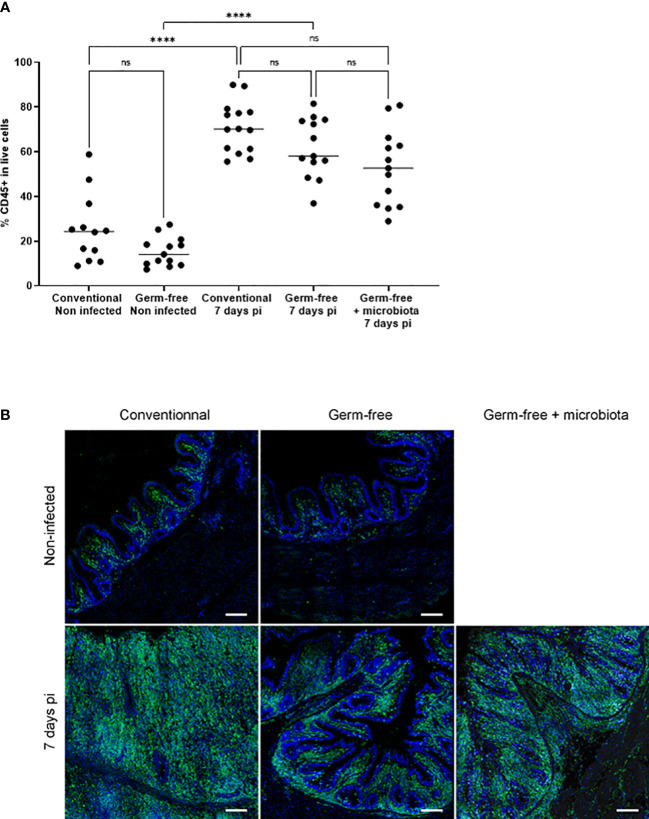
Increase in the percentage of CD45 positive cells in the caecal tissues of infected chickens both germ-free and conventional animals compared to non-infected chickens. Caeca were collected at 7 days pi. Cells were isolated, stained with anti-CD45 antibody, assessed for cell viability (ZombieAqua™) and analysed by flow cytometry **(A)** as described in the materials and methods section. Data are expressed as the median (n ≥ 5/group for at least 2 experiments). Statistical analysis was performed using a Kruskall-Wallis test with a Dunn’s multiple comparisons post-test. ****P < 0.0001; ns: non significant. **(B)** Representative image of CD45^+^ cells recruitment in *E. tenella* infected conventional and germ-free chickens compared to non-infected chickens (SP8 confocal laser-scanning Leica microscope; magnification 63X; image: 2048×2048 pixels, Z stacks and mosaic merge; Leica Application Suite X software).

The presence of microbiota has been shown to induce caecal lesions in the course of *E. tenella* infection and therefore exacerbate mucosal damage. Histological analysis of caecal tissues from infected conventional chickens revealed extensive mucosal alterations ranging from epithelial erosions to ulcers. Conversely, germ-free chickens exhibited fewer and less severe epithelial alterations, primarily erosions. Moreover, introduction of microbiota at day 4 pi led to increased mucosal damage in germ-free chickens, as evidenced by the presence of erosions and/or ulcers, thus confirming its contributory role in the pathology (**Figures 2A, B**). Notably, infected conventional chickens displayed thickened mucosa compared to their non-infected counterparts, and at day 7 pi, mucosal thickness increased in both conventional and germ-free chickens to varying extents likely due to more or less severe inflammatory cell infiltration. The addition of microbiota at day 4 pi appeared to promote cellular infiltration (**Figures 2C**, **3**) and/or local cellular proliferation most probably due to inflammation, ultimately resulting in a partial increase in mucosal thickness in germ-free chickens receiving microbiota comparable to that observed in conventional chickens.

### Inflammatory response in the *E. tenella* infected caeca is strongly enhanced by the presence of microbiota

**Figure 4:**
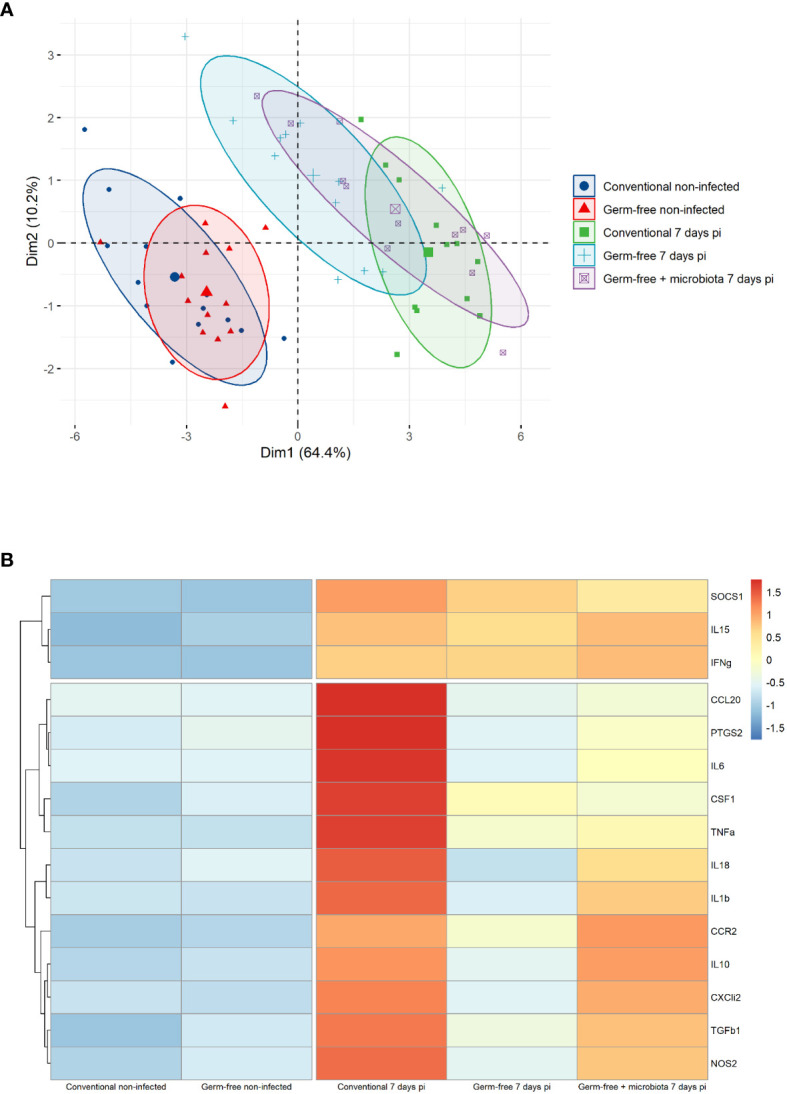
Genes associated to inflammation in germ-free infected chickens are different than the one in conventional infected chickens. **(A)** Principal component analysis (PCA) of RT-qPCR gene expression values from caeca of infected and non-infected chickens following presence or absence of microbiota. Each chicken represented as a dot in specific colour according to its group assignment. Dim 1 (dimension 1) explained 64.4 of the total data variation between animals and Dim 2 (dimension 2) a further 10.2 of the variation. Barycenters for each group are represented in large dots compared to individual values. PERMANOVA test confirmed a significant difference between non-infected and infected chicken clusters. No dissimilarity was observed in conventional and germ-free non-infected groups. At 7 days pi conventional and germ-free clusters were distinct. However germ-free + microbiota cluster was not different from conventional and germ-free infected chickens. **(B)** Z-score hierarchical clustering heatmap depending of median genes expression. Colours represent scaled median values of gene expression with blue for low and red for high values.

**Figure 5:**
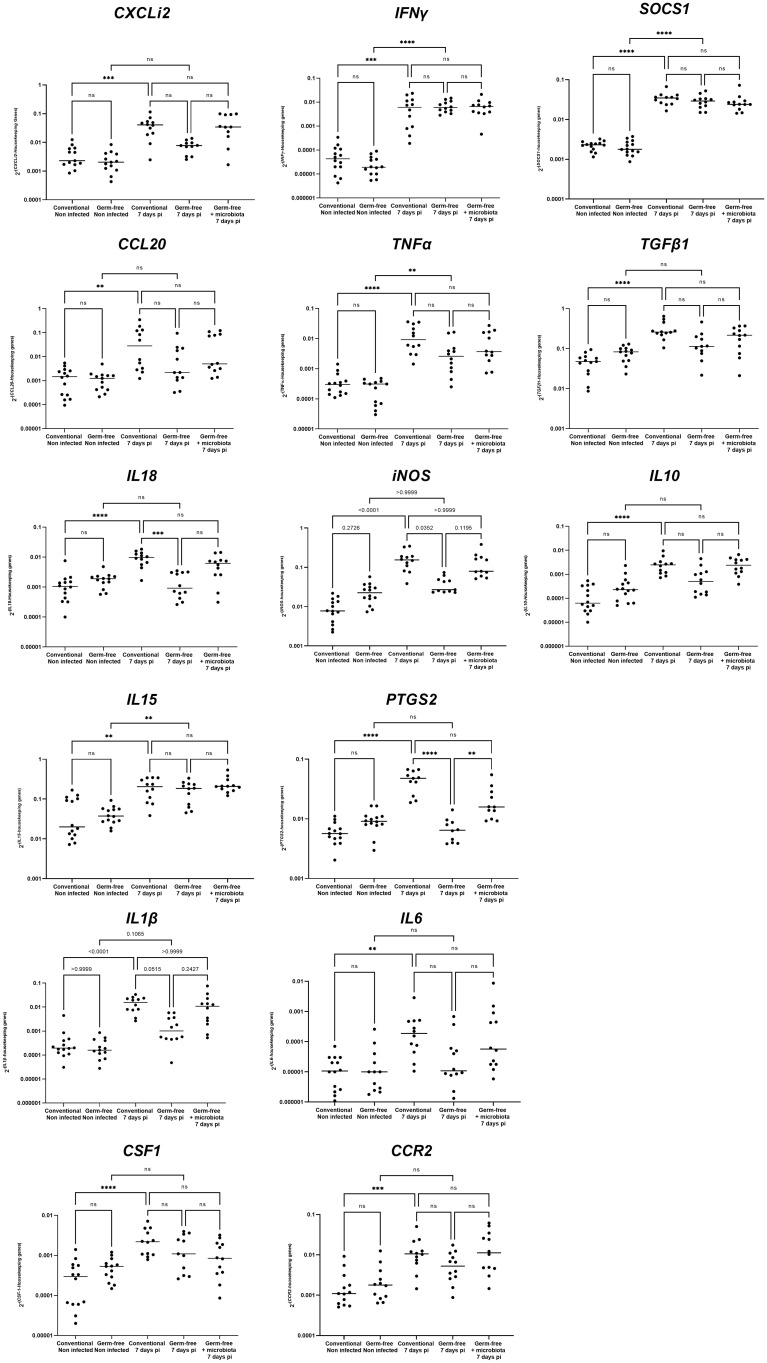
Detailed gene expression assessed by RT-qPCR. Data are expressed as the median. (n ≥ 10/group). Statistical analysis was performed using a Kruskall-Wallis test with a Dunn’s multiple comparisons post-test. **P < 0.01 *** *P* < 0.001 ****P < 0.0001; ns: non significant.

The cell infiltration observed in the caeca of *E. tenella* infected animals was significantly amplified by the presence of microbiota. Through flow cytometry, we identified CD45^+^ cells and found a similar increase in the percentage of total leukocytes in the mucosa of infected conventional and germ-free animals compared to non-infected animals (**Figure 3A**). However, CD45 staining of caecal tissues showed a higher number of total leukocytes in conventional chickens compared to germ-free chickens (**Figure 3B**) likely due to an increased thickness of the caecal mucosa. Gene expression analysis demonstrated that the response to infection differed between conventional and germ-free chickens, with the latter overlapping both groups when supplemented with microbiota (**Figure 4A**); this was confirmed by a PERMANOVA test. Some mediators, such as *IL15*, *IFNγ*, *Suppressor of Cytokine Signaling protein 1 (SOCS1)*, and *TNFα* were increased in response to *E. tenella* in both germ-free and conventional infected chickens, while others, including *IL18*, *Prostaglandin-Endoperoxide Synthase 2 (PTGS2)*, and in a lesser extent *IL1β*, were specifically increased in conventional infected chickens. Additionally*, chemokines (CCL20, CXCLi2)*, *colony stimulating factor 1 (CSF1)*, *Nitric Oxide Synthase 2 (NOS2)* and *IL6* were highly expressed in conventional infected chickens compared to conventional non-infected chickens; however, probably due to variability between animals, the expression of these genes is not significantly different between conventional infected and germ-free infected chickens (**Figures 4B**, **5**). Overall, our results suggest that the presence of microbiota modulates the nature of inflammatory mediators in response to *E. tenella* infection.

### The expression of the tight junction protein *CLDN1* is enhanced by the presence of microbiota in caeca of *E. tenella* infected chickens

**Figure 6:**
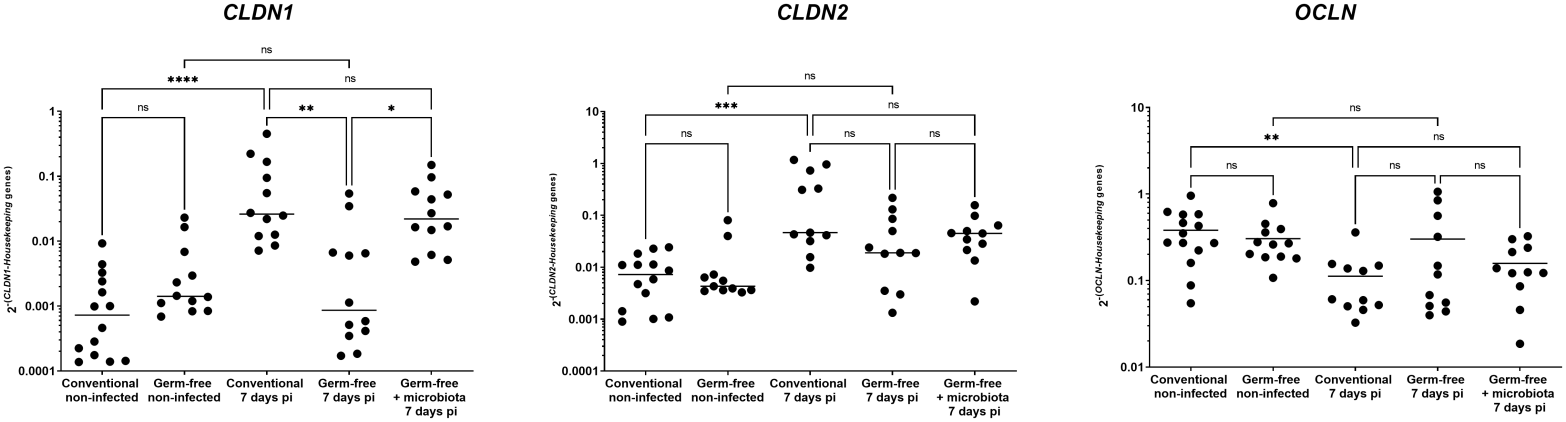
*CLDN1* gene expression increase during infection by *E. tenella* is dependent on the microbiota. Gene expression was assessed by qPCR. *CLDN1* gene expression was increased in conventional chickens at 7 days pi compared to germ-free infected chickens. *CLDN2* gene expression was increased while *OCLN* gene expression was decreased in conventional chickens at 7 days pi compared to conventional non-infected chickens. However, these gene expressions were unchanged in conventional infected chickens compared to germ-free infected chickens due to high variance. Data are expressed as the median (n ≥ 10/group). Statistical analysis was performed using a Kruskall-Wallis test with a Dunn’s multiple comparisons post-test. *P < 0.05 **P < 0.01 *** *P* < 0.001 ****P < 0.0001; ns: non significant.

The presence of microbiota in the caeca of *E. tenella* infected chickens has been shown to enhance the expression of *CLDN1*, a tight junction protein at 5- and 6-days pi (Pham et al., 2021). *E. tenella* infection disrupts the epithelial layer and can cause changes in the expression of tight junction proteins, which are typically regulated by inflammatory cytokines (Andrews et al., 2018). In this study, at day 7 pi, we found that conventional infected chickens had a significant increase in *CLDN1* gene expression compared to conventional non-infected and germ-free infected chickens. Furthermore, the expression of other tight junction protein, such as *CLDN2* and *OCLN* genes, were also significantly altered by the infection in conventional chickens compared to non-infected ones. This effect, however, was not observed in germ-free infected chickens when compared to germ-free non-infected chickens (**Figure 6**). Although there was no significant difference between conventional and germ-free infected chickens due to high data dispersion, these changes in tight junction protein gene expression suggest that the intestinal barrier integrity may be compromised.

### Severe *E. tenella* infection leads to bacteria translocation from the caeca to an extraintestinal site

As previously stated, infection with *E. tenella* results in conspicuous damage to the mucosal lining and substantial alterations in tight junction protein gene expression in conventionally infected chickens, as opposed to germ-free ones. This led to the hypothesis that such mucosal damage may facilitate the translocation from the caeca of commensal bacteria in conventional animals. To investigate this hypothesis, the presence of enterobacteria in the spleen was examined in different models. Interestingly, basal levels of bacterial translocation were observed in non-infected ROSS PM3 chickens, which were undetected in layers (unpublished data), hinting at leaky gut in fast-growing chickens.

**Figure 7:**
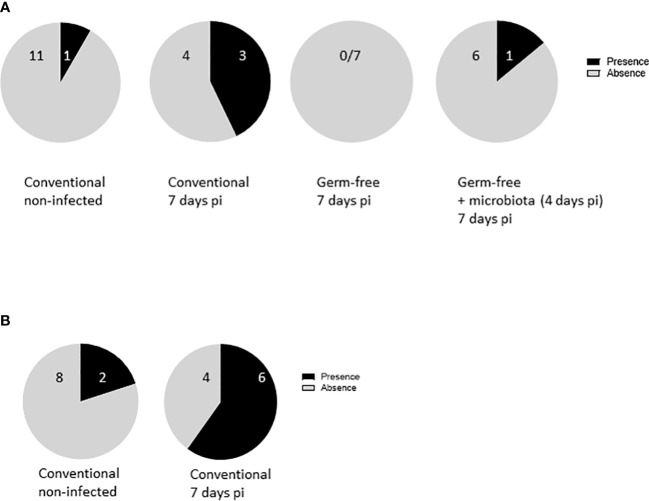
*E. tenella* infection leads to bacterial translocation. **(A)** Commensal enterobacteria were followed in the spleen of infected and non-infected chickens at 7 days pi (n ≥ 7/group). **(B)** APEC were administered to the animals at 1 day old. Chickens were infected with *E. tenella* at 14 days old. Translocation of bacteria in the spleen was performed at day 7 pi (n = 10). Data are expressed as the number of spleens positive or negative for bacteria.

Furthermore, results indicated that bacterial translocation was increased more frequently in conventionally infected chickens (4 out of 7) compared to non-infected ones (1 out of 11). Intriguingly, introducing microbiota to germ-free infected chickens four days pi partially resulted in bacterial translocation (**Figure 7A**) in 1 out of 7 chickens. This correlated well with intermediate epithelial damage, as depicted in histological analysis and with the change in the *CLDN1* gene expression with the infection.

**Figure 8:**
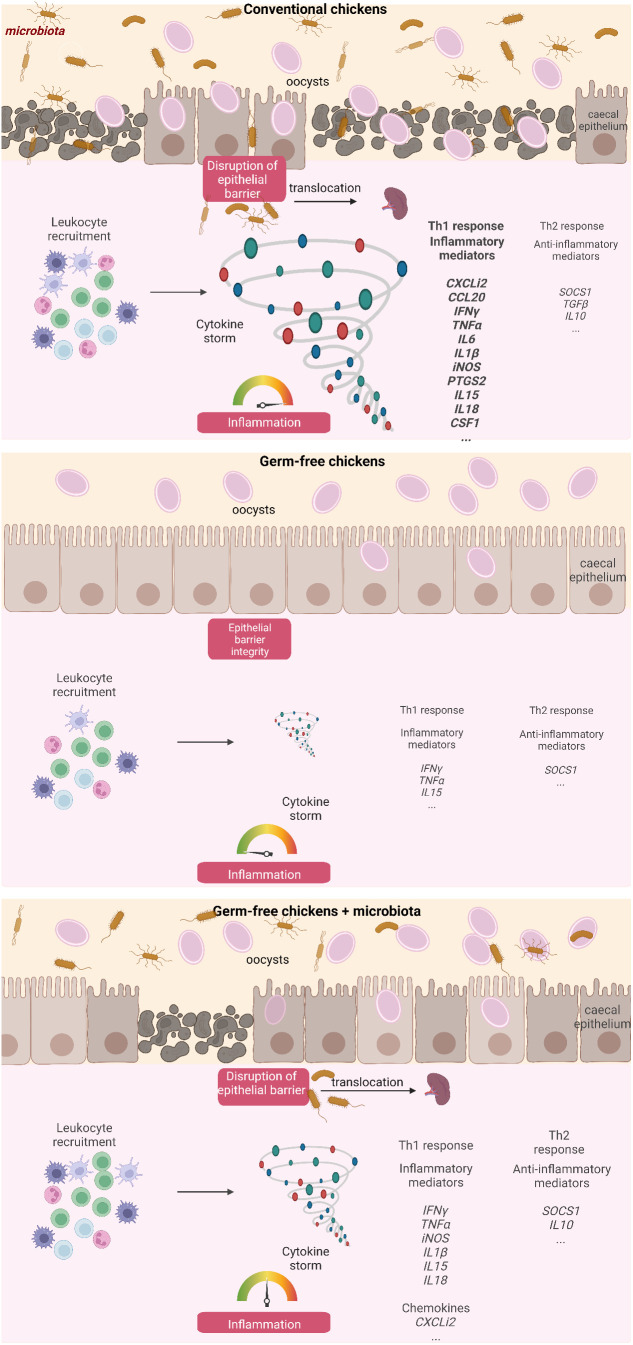
Summary diagram describing the influence of the caecal microbiota on the development of inflammation and the loss of intestinal integrity at day 7 pi with *E. tenella*. Created with BioRender.com (JV25NHUMTD, FE257PUXU1, RV257PUXXN).

To validate these findings, APEC was administered to chicks at 7 day old, followed by *E. tenella* infection at 14 day old. Spleens were collected from both infected and non-infected chickens, and bacteriological analysis was performed for the presence of APEC. After *E. tenella* infection, there was a conspicuous increase in APEC presence in the spleens of conventionally infected chickens (4 out of 6) compared to conventionally non-infected ones (2 out of 8), pointing to a potential bacterial translocation resulting from the infection (**Figure 7B**). These data support the notion that epithelial damage in conventionally infected chickens presents an opportunity for bacterial translocation, ultimately leading to an exaggerated inflammatory response, as opposed to germ-free ones (**Figure 8**).

## Discussion

The present study has shed light on the pivotal role that the microbiota plays in the physiopathology of *E. tenella* infection, as evidenced by higher levels of caecal lesions and inflammation in conventional chickens compared to their germ-free counterparts. Moreover, the presence of microbiota during infection led to disruption of the epithelial layer, causing bacterial translocation and potentially resulting in opportunistic infections.

At homeostasis, the microbiota confers manifold benefits to the immune system by providing metabolites such as short chain fatty acids, which are derived from alimentary fibers. These metabolites promote the maturation of the innate immune system, especially at an early age, and foster the development of the intestinal barrier structure and function, thus shielding the host from potential pathogen entry (Hayes et al., 2018). However, when this balance is perturbed, as in the case of a severe infection, the microbiota’s role can turn deleterious for the host (Belkaid and Hand, 2014). In this study, we investigated the impact of the microbiota on the physiopathology of *E. tenella* infection. Conventional and germ-free chickens were then infected with *E. tenella* for 7 days and microbiota from conventional chickens was administered to germ-free chickens at 4 days pi. To compare the physiopathology of infected with non-infected conditions, non-infected germ-free and conventional chickens were used. In the present study, conventional microbiota was not administered to non-infected germ-free chickens for 3 days. Even tough an effect of the microbiota in non-infected germ-free animals cannot be rule out, as we observed no differences in the parameters measured in non-infected germ-free and conventional chickens, it is unlikely to observe a different response in non-infected germ-free chickens receiving a microbiota. Previous research revealed that the microbiota plays a role in parasite development, as evidenced by lower parasite loads in germ-free chickens compared to conventional chickens, despite similar invasion rates in host epithelial cells (Gaboriaud et al., 2020). In this latter study, we showed that a high dose of inoculum of 10 000 oocysts with the INRAE *E tenella* strain led to a similar parasite load in conventional and germ-free chickens at day 7 post-infection. In the present work, we chose this high dose of inoculum and confirmed a similar parasite load between groups. This allowed us to investigate the role of microbiota on the physiopathology of the infection independently of the parasite load, which also contributes to the pathology by inducing mechanical disruption of epithelial cells. We first assessed lesion scores based on several parameters, such as the presence of petechiae, the thickening of the caecal wall, the aspect of the caecal content (presence of blood or caseous core), distension of the caecal wall with its content, or death of the animal (Johnson and Reid, 1970). Under our experimental conditions, we observed markedly lower lesion scores in germ-free chickens, which did not reach the levels seen in conventional chickens even at a very high inoculum dose (50 000 oocysts). Additionally, the microbiota transplantation from healthy conventional chickens into germ-free chickens at 4 days pi led to a significant increase in lesions. Our findings underscore the critical role of microbiota in the formation of lesions during *E. tenella* infection. Undoubtedly, an elevated inoculation dose of the parasite corresponds to a greater degree of lesion scores even in germ-free chickens, indicating that not only the microbiota, but also the replication of the parasite and the ensuing inflammation associated with the infection are involved in the genesis of lesions. Interestingly, the physiopathology of the infection *E. acervulina* appears to be unchanged by microbiota, as germ-free chickens infected with *E. acervulina* demonstrate comparable clinical and biochemical parameters (namely, weight loss, serum protein and lipid changes) to the conventional ones (Lafont JP et al, 1975). Although the parasite load and the lesion scores were not determined in that study, the differences between the physiopathology of the two species could potentially be explained by the considerably higher bacterial load and diversity present in the caeca as compared to the duodenum. Furthermore, the nature of bacteria residing in these two compartments varies, with the caecal microbiota composition and complexity being greater than that of the duodenum, which mostly contains *Lactobacilli*. In the caeca, nearly 1000 diverse species belong to two major phyla, namely, *Bacillota* (former name Firmicutes) and *Bacteroidota* (former name Bacteroidetes), along with two minor phyla, namely, *Actinomycetota*) and *Pseudomonadota* (former name Proteobacteria) (Rychlik, 2020). Consequently, we contend that the discrepancies in microbiota composition may account for differences in the clinical symptoms between *Eimeria* species.

A prior histological investigation has indicated that in conventional chickens, the mucosa thickens as a result of an infiltration of inflammatory cells, including macrophages, heterophils, and lymphocytes (Lillehoj and Trout, 1996). The present study demonstrates that a thickening of the mucosa and recruitment of leukocytes occurs in infected conventional chickens, as well as to a lesser extent in germ-free infected chickens, potentially due to a lower or different inflammatory response. Supporting this observation, the gene expression profile of inflammatory and anti-inflammatory mediators shows a distinct pattern between germ-free and conventional infected chickens. In both types of infected chickens, transcription of only certain cytokines, including *IL15*, *IFNγ*, *TNFα*, and the anti-inflammatory mediator *SOCS1*, increase, indicating that inflammation also occurs in germ-free infected chickens. Similar to previous findings in conventional animals (Laurent et al., 2001; Hong et al., 2006), a significant increase in the expression of genes related to inflammation (*CXCLi2*, *CCL20*, *IL18*, *CSF1*, *iNOS*, *PTGS2*, *IL1β*, *IL6*, *CCR2*) and the anti-inflammatory (*TGFβ1* and *IL10*) response is observed with *E. tenella* infection. However, the expression of these genes is not significantly increased in germ-free infected chickens compared to non-infected germ-free chickens. Notably, *IL18*, *PTGS2*, and, to a lesser extent, *IL1β* have significantly higher expressions in infected conventional compared to infected germ-free chickens. IL18 and IL1β are potent pro-inflammatory cytokines (Hua et al., 2015; Venuprasad and Theiss, 2021) while *PTGS2* is induced by inflammatory stimuli such as IL1β and TNFα but also by bacterial components and is involved in the regulation of NLRP3 inflammasome (Hua et al., 2015). The activation of the inflammasome pathway can lead to the activation and release of pro-inflammatory cytokines such as IL18 and IL1β, which, in a murine model of infection by *Cryptosporidium tizzeri*, have been shown to promote the production of IFNγ, known to play a crucial role in parasite control (Sateriale et al., 2021). Similarly, in *E. tenella* infection, IFNγ has been shown to inhibit its replication (Heriveau et al., 2000). Given the known role of IFNγ in parasite control, the similarity in its expression between the two models implies that IFNγ may be similarly effective in controlling *E. tenella* replication in both conventional and germ-free animals. Overall, our data suggest that in *E. tenella* infection, the microbiota may induce the NLRP3 inflammasome, participating to the cleavage of procaspase 1 and subsequent leads to activation and release of pro-inflammatory cytokines IL1β and IL18. Thus, it is possible that in *E. tenella* infection in the presence of microbiota, bacterial moieties and/or metabolites may potentiate the epithelial cell response such as IL1β and IL18 production leading to an amplified inflammatory cascade in the caecal tissue with the involvement of different mediators by various type of leukocytes.

The intestinal epithelial layer protects the organism against the entry of potential pathogens and is preserved with the maintenance of junction proteins present between epithelial cells. Modification of the tight junction protein expression by inflammatory cytokines can then alter the intestinal permeability (Andrews et al., 2018). In the neonatal model of infection with the apicomplexan parasite, *Cryptosporidium parvum*, we showed that increased permeability was the result of both the parasite on the adherens junctions proteins E-cadherin and β-catenin and the release of inflammatory cytokines (TNFα and IL1β) by inflammatory monocytes (De Sablet et al., 2016). In the present study, in conventional chickens, severe *E. tenella* infection led to an acute inflammation, the disruption of the epithelial layer, an alteration of tight junction protein gene expression more particularly with an increase in *CLDN1* gene expression and a higher frequency of bacterial translocation. Overall, in severe *E. tenella* infection and in the presence of microbiota, the acute inflammatory response may modify the expression of tight junction proteins resulting in an increase in intestinal permeability and to potential bacterial translocation.

Recent studies using mouse models have demonstrated the crucial role of commensal bacteria in host defense against various pathogenic infections caused by bacteria, fungi, and parasites. The absence of microbiota has been shown to increase susceptibility to infections caused by *Listeria monocytogenes*, *Salmonella* Typhimurium, *Klebsiella pneumoniae*, and *Cryptococcus gatti*, indicating a protective role of commensal bacteria in host defense (Nardi et al., 1989; Fagundes et al., 2012; Mittrucker et al., 2014; Costa et al., 2016). This protection may be attributed to the competition between pathogenic and commensal bacteria, limiting the growth and colonization of pathogenic bacteria. However, in some cases, the microbiota may exacerbate inflammation, tissue damage, and promote pathogen transmission. For instance, the interaction between the microbiota and *Trichuris muris* is necessary for parasite egg hatching and subsequent transmission (Hayes et al., 2010). In the case of *Toxoplasma gondii* infection, germ-free mice were found to have less inflammation and intestinal permeability than conventionally-raised mice, suggesting the contribution of the microbiota to an exaggerated inflammatory response (Nascimento et al., 2017). A similar deleterious role of Gram-positive bacteria was observed in *E. falciformis*, that developed also in the caecum of mice. Indeed, antibiotic administration depleting Gram-positive bacteria reduced histopathological scores and oocyst excretion (Gong et al., 2022). In addition, in a mouse model of dextran sodium sulfate-induced colitis, Gram-positive bacteria were responsible for the recruitment of phagocytes and enhanced inflammation, indicating a potential harmful role of commensal bacteria (Nakanishi et al., 2015). Further studies are needed to investigate the role of the microbiota in *E. tenella* infection in chickens.

The contribution of the microbiota to host defense against infection is complex and context-dependent, and further studies are needed to understand the underlying mechanisms. In the context of acute inflammation, it is not uncommon to observe a dysbiosis marked by an increase in bacterial populations that can potentially exacerbate inflammation and tissue damage. In *Eimeria* infection, an increase in Proteobacteria and a decrease in Firmicutes has been noted (Macdonald et al., 2017). This shift towards a dominance of Proteobacteria has been shown to be associated with more severe physiopathology due to the translocation of these bacteria, which can contribute to an exacerbation of inflammation by promoting differentiation and activation of cells towards an inflammatory phenotype (Belkaid and Hand, 2014). To address this issue, it is hypothesized that replenishing Firmicutes to levels and diversity comparable to non-infected animals or administering bacterial probiotics may help to attenuate inflammation and its adverse effects on the integrity of the intestinal barrier. Further research is needed to validate these hypotheses and identify potential molecular mechanisms underlying these effects.

In conclusion, microbiota has revealed its crucial impact in the physiopathology of *E. tenella* infection. The microbiota is responsible for inducing acute inflammation and extensive lesions in the caeca, leading to bacterial translocation and secondary opportunistic infections. The identification of key microbiota bacteria responsible for the pathology of coccidiosis can aid in the development of alternative strategies for managing and preventing this infection in the poultry industry. Early age administration, dietary modification, and prebiotic use could be potential methods for modulating the microbiota and improving the outcomes of coccidiosis in poultry. Therefore, further research in this field may pave the way for more effective management strategies and improved animal welfare in the poultry industry.

## Materials and methods

### Ethical statement

Animal experiments were approved by the French legislation (Décret: 2001‐464 29/05/01), the EEC regulation (86/609/CEE) and by the ethics committee of Centre Val de Loire (CEEA VdL n°19): 2018‐04‐26 (APAFIS N°13904).

### Animals

Hatching and growth of conventional and germ-free Ross PM3 broilers were performed in the facility for Infection of Farm, Model and Wildlife Animals (PFIE, Centre INRAE Val De Loire: https://doi.org/10.15454/1.5572352821559333E12; member of the National Infrastructure EMERG’IN). Ross PM3 eggs were obtained from French farms, decontaminated with a 1.5% Divosan Plus (VT53, Johnson Diversey, France). Eggs were incubated, decontaminated a second time. Animals were hatched in an incubator for conventional chicks or an isolator for germ-free animals. The absence of bacteria was confirmed in germ-free chicks; and the development of a microbiota was present in conventional chickens (Guitton et al., 2020).

### Infection

Oral infection was performed on two-week-old broilers with 10 000 sporulated oocysts PAPt36 strain (*Et*-INRAE). At day 7 pi, chickens were euthanized using electronarcosis. Caeca were collected and lesion scoring performed as described by (Johnson and Reid, 1970). After washing the caeca, the tissue was fixed in 4% formaldehyde (Laurypath, Chaponost, France) for histological analysis or kept on ice for flow cytometry or directly frozen in liquid nitrogen and stored at -80°C for transcriptomic analyses. Spleens were collected for further bacteriological analysis.

### Bacterial translocation

For bacterial translocation study, the avian pathogenic *Escherichia coli* (*E. coli*) strain BEN2908, O2:K1:H5 ST95, a nalidixic acid-resistant derivative of strain MT78 isolated from the trachea of a chicken with a respiratory infection (Dho and Lafont, 1982) was used. This strain is able to colonize the intestine at high level even in the presence of others *E. coli* strains of the microbiota (Porcheron et al., 2012). An overnight culture in LB-Miller medium at 37°C with agitation was diluted in sterile apyrogenic DPBS (Sigma Aldrich) at a final concentration of 5×10^4^ cfu/mL. Chickens were orally inoculated at one day old with 200 µl of the bacterial suspension. Intestinal colonization of BEN2908 was assessed by plating serial dilutions of feces onto Drigalski agar plates (Bio-Rad, Hercules, CA, USA) supplemented with nalidixic acid at 30 µg/ml. To assess the systemic dissemination of the APEC strain and enterobacteria at day 7 pi, animals were euthanized, spleens were collected, weighted and homogenized in 3.5 mL of sterile DPBS using gentleMACS™ Dissociator (Miltenyi Biotec, Bergisch Gladbach, Germany). Serial dilutions were plated onto Drigalski agar plates (Bio-Rad) supplemented with nalidixic acid at 30 µg/mL (selection of BEN2908) or without antibiotic (selection of enterobacteria and BEN2908) for bacterial quantification.

### Cell isolation, staining and flow cytometry analysis

For analysing the percentage of leukocytes in the caeca, tissues were scraped and cells were dissociated in a DMEM-F12 medium supplemented with collagenase H (1 mg/ml, Roche Basel, Switzerland) for 30 min at 37°C under stirring. After washing and filtering, viable caecal cells were counted using Trypan blue and 2 × 10^6^ live cells were harvested for staining. First, cells were incubated for 10 min at 4°C with PBS supplemented with 2% naïve chicken serum and 2 mM EDTA for blocking unspecific staining. Then, cells were stained with anti-CD45 antibody labelled with APC for leukocytes (SouthernBiotech, Birmingham, AL, USA) and ZombieAqua™ for assessing cell viability (BioLegend, San Diego, CA, USA) in PBS supplemented with 2% FBS and 2 mM EDTA for 30 min at 4°C. Cells were washed, fixed with fixation buffer (BD Biosciences, Franklin Lakes, NJ, USA) and filtered before flow cytometry analysis (LSR Fortessa X-20, BD Biosciences). Debris were excluded and only viable cells were selected. In this selected region, CD45-positive cells were assessed and represented as a percentage of live cells.

### Histological analysis

Tissues fixed in formaldehyde (Laurypath) were embedded in paraffin wax, sliced and stained with Hemalun Eosin Saffron.

### Immunofluorescence staining

Caecal tissues were directly frozen in OCT. Tissues were sliced and fixed in ice cold 50% ethanol, 50% acetone. Leukocytes were stained for 1 h at 37°C using anti-anti-CD45 Alexa Fluor 488 (SouthernBiotech). Cell nuclei were counterstained with DAPI (ThermoFisher Scientific, Waltham, MA, USA) for 5 min at room temperature and mounted using Permafluor™ (Epredia, Kalamazoo, MI, USA). Stack of tiled images were acquired with SP8 confocal laser-scanning microscope (HC PL APO CS2 63x/1.20 WATER; 2048 × 2048 pixels; Leica Application Suite X software (3.7.4.23463 version) and a mosaic merge was performed to obtain wide images of the caecal tissue slices.

### Gene expression analysis

Gene expression analysis was performed as described previously (Gaboriaud et al., 2020). Briefly, caecal tissues total RNA was extracted using TRIzol (Life Technologies, Carlsbad, CA, USA) and cDNA was synthetized using M-MLV Reverse Transcriptase (Promega, Madison, WI, USA). qPCRs were realised on CFX96 Touch Real-Time PCR Detection System (Bio-Rad) using iQ™ SYBR^®^ Green Supermix (Bio-Rad). Inflammatory, anti-inflammatory and protein junction gene expressions were analysed using *Gallus gallus* housekeeping genes *G10* and *GAPDH* (Supplementary data: **Table 1**, Eurogentec, Seraing, Belgium). The protocol used for qPCR was: 95°C for 5 min and 40 cycles at 95°C for 10 s and 60°C for 15 s followed by 60°C for 5 s. Melting curves were performed at 60°C for 5 s followed by gradual heating (0.5°C/s) to 95°C. qPCRs were performed in duplicate for each experiment. Gene expression was normalised to Ct values obtained for *Gallus gallus G10* and *GAPDH* using the formula: 2^-(Ct^ *^Gallus gallus specific gene^* ^– Ct^ *^mean of Gallus gallus housekeeping genes^* ^)^. Gene expression values are expressed as the median. To obtain a global view of inflammatory gene expression, a heatmap analysis was performed using the R package Pheatmap (Kolde, 2019). Median of gene expression values were normalized with a Z-score approach and scaled by genes. Hierarchical clustering analysis was also performed according to Z-score values. To investigate if gene expression depends on the animal group, a Principal Component Analysis (PCA) was performed. 2^-dCT^ values were transformed in log10, quality of variables based on cos^2^ was established and PCA was generated using the R package Factominer (Lê et al, 2008).

**Table 1:** Sequences of primers used for RT-qPCR.

Gene	Accession number	Forward sequence (5’ to 3’)	Reverse sequence (5’ to 3’)
*IL8L2 (CXCLi2)*	NM_205498	GCTCTGTCGCAAGGTAGGAC	GGCCATAAGTGCCTTTACGA
*CCL20*	NM_204438	GGCACAAAGCAACCAAGATT	GGATTTACGCAGGCTTTCAG
*IL18*	NM_204608	CACTGTTACAAAACCACCGC	CTTAAAAGCCTTGGAGCTGC
*IL15*	NM_204571.2	CTTGTCCATAGGTTTCCGAG	GAGTTTTGTGTTGGCTGTGC
*CSF1*	NM_001389459	GCGACTCTGTCTGCTACGTG	CGAAGGTCTCCTTGTTCTGC
*IFNγ*	NM_205149.2	TGGCGTGAAGAAGGTGAAAGA	TCTGAGACTGGCTCCTTTTCCT
*TNFα*	MF000729	CGCTCAGAACGACGTCAA	GTCGTCCACACCAACGAG
*NOS2* *(iNOS)*	NM_204961.2	GCGTGTCCTTTCAACGGCT	CCAGTCCCATTCTTCTTCCA
*PTGS2*	NM_001167718.2	CTGCTCCCTCCCATGTCAGA	CACGTGAAGAATTCCGGTGTT
*IL1β*	NM_204524.2	AGGCTCAACATTGCGCTGTA	CTTGTAGCCCTTGATGCCCA
*IL6*	NM_204628	GCTTCGACGAGGAGAAATGC	GCCAGGTGCTTTGTGCTGTA
*CCR2*	NM_001045835.1	ATGCCAACAACAACGTTTGA	TGTTGCCTATGAAGCCAAA
*SOCS1*	NM_001137648.1	CACGCACTTCCGAACCTTTC	ACTTCAGCTTCTCATGGGCG
*TGFβ1*	NM_001318456	AGGATCTGCAGTGGAAGTGGAT	CCCCGGGTTGTGTTGGT
*IL10*	NM_001004414	CATGCTGCTGGGCCTGAA	CGTCTCCTTGATCTGCTTGAT
*GAPDH*	NM_204305	CCACAACATACTCAGCACCTGC	GTCCTCTGGCAAAGTCCAAG
*G10 (BUD31)*	416492	AACAGCCTCTGCATCCACAGT	TCAAGGAAGGGTACGCTGACA

Sequences of primers for genes coding for protein junctions are described in (Gloux et al., 2019).

### Statistical analysis

For all data except PCA, statistical analysis was performed using GraphPad Prism^®^ 9 (GraphPad Software Inc., La Jolla, CA, USA). For two groups, a non-parametric Mann-Whitney test was used. For more than two groups, a Kruskall-Wallis test with a Dunn’s multiple comparisons post-test was performed. For PCA, statistical differences between groups were performed using a pairwise PERMANOVA (Permutational multivariate analysis of variance) test (Anderson, 2001) with 999 permutations *via* Vegan (Jari et al, 2022) and pairwiseAdonis R packages (Martinez Arbizu, 2017). All packages were performed through Rstudio software version 4.1.0 (RStudio: Integrated Development for R. RStudio, PBC, Boston).

## Data availability statement

The original contributions presented in the study are included in the article/**Supplementary Material**. Further inquiries can be directed to the corresponding author.

## Ethics statement

The animal study was approved by ethics committee of Centre Val de Loire (CEEA VdL n°19). The study was conducted in accordance with the local legislation and institutional requirements.

## Author contributions

FB designed the experiments. PG, GS, FT, NL, LS, and FB performed the experiments. EG provided the conventional and germ-free chickens. TL and ML performed the histology staining on caecal tissues and the analysis. CR and GS performed the immunofluorescence staining and confocal microscopy acquisition. PG, GS, and FT performed the cell staining and flow cytometry analysis. PG, GS, FT, and LS performed the qPCR and analysed the data. FT performed the PCA and Heatmap analysis. CS and NL realised the bacteriological controls and bacteriological analysis in the spleens. FB, PG, GS, and FT analysed the data. FB, CS, FL, EG and RG obtained funding. FB, PG, GS, FT, SL-L, FL, RG, and CS discussed the data. FB, GS, FT, AS, SL-L, FL, RG, and CS, wrote and/or reviewed the manuscript. All authors approved the final version of the manuscript. All authors contributed to the article.

## Supplementary material

Supplementary Figure 1 Oocysts load in caecal contents at 7 days pi. Conventional and germ-free chickens were orally infected with 10 000 oocysts of *E. tenella*. Oocyst load in caecal contents was evaluated at day 7 pi. Medians are represented (Kruskall-Wallis test with a Dunn’s multiple comparisons post-test; n ≥ 11).
