# Supplementary figures and images for: The caecal microbiota promotes the acute inflammatory response and the loss of the intestinal barrier integrity during severe Eimeria tenella infection
Source: Front Cell Infect Microbiol. 2023 Aug 23;13:1250080. doi: 10.3389/fcimb.2023.1250080 (PMC10482108; doi:10.3389/fcimb.2023.1250080)

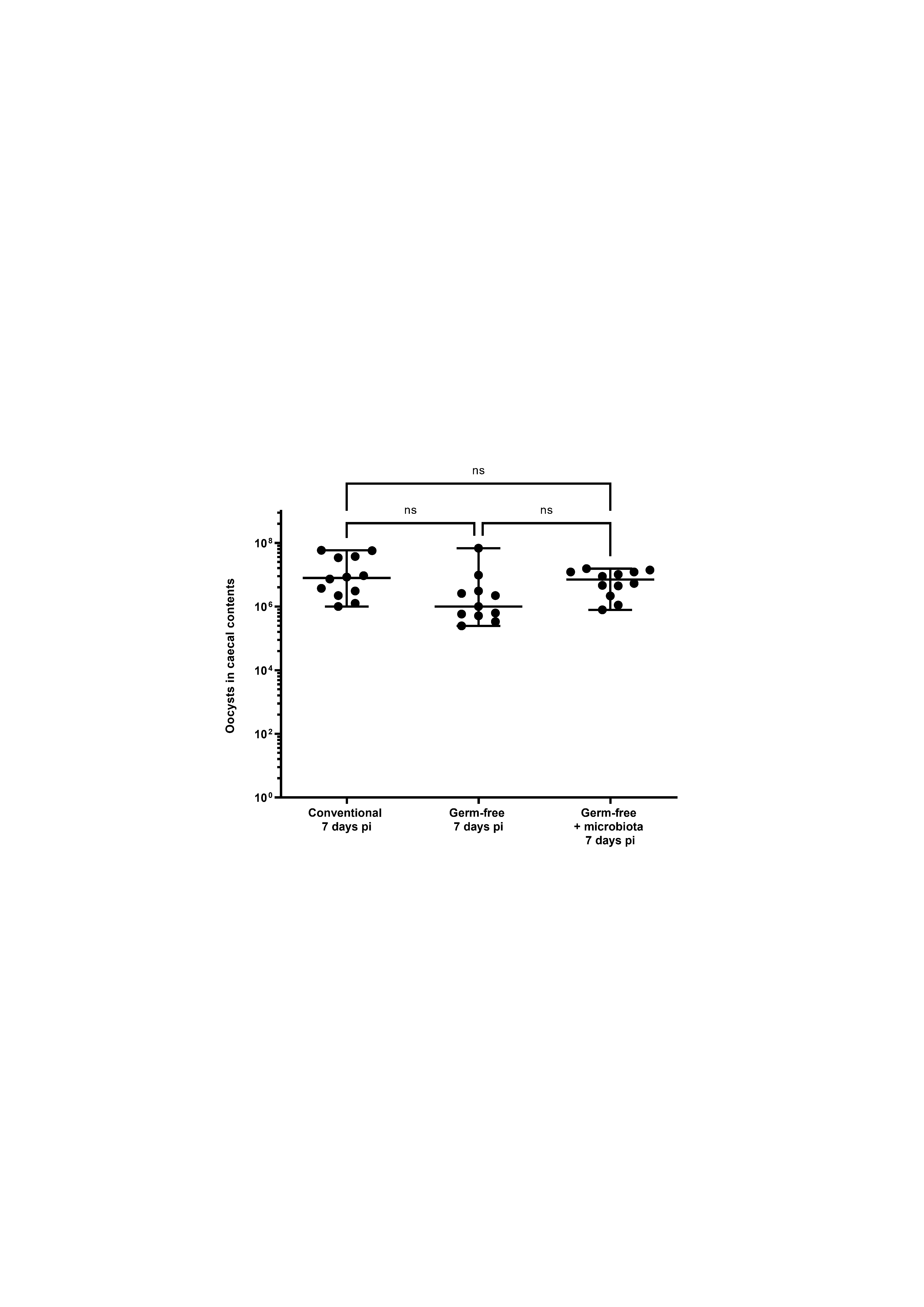

Supplement: Supplementary Figure 1 — Oocysts load in caecal contents at 7 days pi. Conventional and germ-free chickens were orally infected with 10 000 oocysts of E. tenella. Oocyst load in caecal contents was evaluated at day 7 pi. Medians are represented (Kruskall-Wallis test with a Dunn’s multiple comparisons post-test; n ≥ 11). [file Image_1.tif]
